# Enhanced Resolution of Evolution and Phylogeny of the Moths Inferred from Nineteen Mitochondrial Genomes

**DOI:** 10.3390/genes13091634

**Published:** 2022-09-12

**Authors:** Xiaofeng Zheng, Rusong Zhang, Bisong Yue, Yongjie Wu, Nan Yang, Chuang Zhou

**Affiliations:** 1 Key Laboratory of Bioresources and Ecoenvironment (Ministry of Education), College of Life Sciences, Sichuan University, Chengdu 610064, China; 2 Institute of Qinghai-Tibetan Plateau, Southwest Minzu University, Chengdu 610064, China; 3 Collaborative Innovation Center for Ecological Animal Husbandry of Qinghai-Tibetan Plateau, Southwest Minzu University, Chengdu 610064, China

**Keywords:** moths, mitochondrial genome, evolutionary rate, overlap, intergenic spacer, phylogeny

## Abstract

The vast majority (approximately 90%) of Lepidoptera species belong to moths whose phylogeny has been widely discussed and highly controversial. For the further understanding of phylogenetic relationships of moths, nineteen nearly complete mitochondrial genomes (mitogenomes) of moths involved in six major lineages were sequenced and characterized. These mitogenomes ranged from 15,177 bp (*Cyclidia fractifasciata*) to 15,749 bp (*Ophthalmitis albosignaria*) in length, comprising of the core 37 mitochondrial genes (13 protein-coding genes (PCGs) + 22 tRNAs + two rRNAs) and an incomplete control region. The order and orientation of genes showed the same pattern and the gene order of *trnM-trnI-trnQ* showed a typical rearrangement of Lepidoptera compared with the ancestral order of *trnI-trnQ-trnM*. Among these 13 PCGs, *ATP8* exhibited the fastest evolutionary rate, and Drepanidae showed the highest average evolutionary rate among six families involved in 66 species. The phylogenetic analyses based on the dataset of 13 PCGs suggested the relationship of (Notodontidae + (Noctuidae + Erebidae)) + (Geometridae + (Sphingidae + Drepanidae)), which suggested a slightly different pattern from previous studies. Most groups were well defined in the subfamily level except Erebidae, which was not fully consistent across bayesian and maximum likelihood methods. Several formerly unassigned tribes of Geometridae were suggested based on mitogenome sequences despite a not very strong support in partial nodes. The study of mitogenomes of these moths can provide fundamental information of mitogenome architecture, and the phylogenetic position of moths, and contributes to further phylogeographical studies and the biological control of pests.

## 1. Introduction

Lepidoptera (butterflies and moths) is the largest single radiation of plant-feeding insects, the diversification of which is presumed to be in synchrony with angiosperms insects [1,2]. Lepidoptera insects cover 133 families, 43 superfamilies and 157,424 extant species in the world, and moths are estimated to make up nearly 90% of the diversity of species [3]. Moths have a profound impact on human society whether acting as major pests in agriculture and forestry [4]; or working as pollinators and economic insects [5,6]; or undertaking as model systems for the studies of genetics, physiology, development, ecology, and evolutionary biology [7,8].

Researches on phylogenetic relationships of moths have been carried out extensively, especially on some large families like Erebidae [9,10], Noctuidae [11,12,13,14,15,16] and Geometridae [17,18,19,20], among which some remarkable and fundamental works were achieved by Kristensen et al. [21,22] and Minet et al. [23,24] initially with the studies of morphological and anatomical characteristics. As molecular markers widely applied to phylogenetic studies, new evidence has been consistently presented based on mitochondrial genes, nuclear ribosomal DNA, nuclear protein-coding genes or a combined method [25,26,27,28,29]. For Noctuoidea, the largest superfamily of moths, its monophyly was morphologically supported by the unique apomorphic character with a metathoracic tympanal organ, and also strongly confirmed by various molecular datasets [25,27,29]. On the other hand, the phylogenic conflicts within Noctuoidea between morphological and molecular results were often reported. Contrasting with the traditional ten-family or the later revised five-family classification system [13,30], recently, a new six-family classification system of Noctuoidea has been well accepted using one mitochondrial gene *(COI*) and seven nuclear genes (*EF-1α*, *wingless*, *RpS5*, *IDH*, *MDH*, *GAPDH* and *CAD*): Oenosandridae, Notodontidae, Erebidae, Euteliidae, Nolidae and Noctuida [29]. The Doidae showed a close relationship with groups of Drepanoidea based on molecular support [25,27] instead of an affiliation with Noctuoidea traditionally [22,31]. Noctuidae in the broad former sense was paraphyletic including Lymantriidae and Arctiidae [22], but recently these two have been subsumed into Erebidae as subfamily concepts [10,29]. Similarly, some members of Drepanidea were related with Geometroidea for mostly shared features of abdominal tympanal organs [24,32,33,34]. However, inferred from *EF-1a* and *COI* sequences, Drepanidea consisted of the sole family Drepanoidae (including four subfamilies: Drepaninae, Oretinae, Thyatirinae and Cyclidiinae) and the former Epicopeiidae was suggested to be excluded as a separate superfamily [34]. Although the majority of divergences within moths seem credibly established, there remains broadly weak support or unstable nodes among partial subordinate taxa because of deficient genetic information or sparse sampling, thus resulting in conflicting results [3]. Short internal branches resulting from rapid radiation also increase the difficulty of the phylogeny resolution [35].

The mitochondrial genome has been considered as an ideal tool in studies of comparative and evolutionary genomics, molecular evolution, phylogenetics and population genetics [36,37,38] regarding its simple structure, maternal inheritance and that it rarely undergoes recombination [39,40,41]. The insect mitogenomes are a compact circular molecule with a length of 15–18 kb consisting of 37 conserved genes (13 protein-coding genes (PCGs) + 22 tRNA + 2 rRNA), which have been extensively studied, involved in all orders [39]. Recently, multiple studies have used them to address phylogenetic questions about moths where multi-gene analyses have been either unresolved or poorly supported. Some novel findings are consistently proposed with mitogenome data such as the polyphyletic macrolepidopteran superfamilies [42,43,44], which have never been reported in morphological analyses [24,45] or in combined datasets of nucleotide sequences and morphological characteristics [46]. Given the rapid developing sequencing technique, fast accumulated mitogenome resources of moths would accelerate our understanding of the phylogeny, genetics and evolution of moths [47,48]. Though RNA-Seq [35] and whole-genome sequences [49] are also being used in phylogenetic studies of insects, they are limited to few taxa samplings and expensive bills.

In this study, we sequenced nearly complete mitochondrial genomes of nineteen moths across six major families of Lepidoptera (including two of Drepanidae, three of Erebidae, six of Geometridae, one of Noctuidae, four of Notodontidae and three of Sphingidae) based on next-generation sequencing. Comparative analyses on characteristics of mitochondrial genomes and phylogenetic investigations on these species were performed with extensive taxon sampling involved in 66 species in total, with an attempt to provide insight into the evolution of those major lineages of moths. Due to the small taxa sampling, we did not expect a robust phylogenetic tree, but focused more on the impact of newly sequenced species on the phylogenetic classifications compared with previous studies.

## 2. Materials and Methods

### 2.1. Taxon Sampling

All these 19 specimens were collected from Mount Qingcheng, Sichuan Province, China. The samples were initially placed in 100% ethyl alcohol under −20 °C in the lab until DNA extraction (College of Life Sciences, Sichuan University). We initially identified all the samples through traditional morphological keys [50,51]. For further verifying the morphological identification, we performed blast searches of the nucleotide collection (nr/nt) database of the NCBI based on cytochrome c oxidase I (*COI*) mitochondrial gene. Total genomic DNA was extracted by DNeasy Blood & Tissue kit following manufacturer instructions, and the quality of total DNA was checked with 1% agarose gels. The partial sequences of *COI* gene (~630 bp) for each species served as DNA barcode were amplified with the primers LC01490: 5′-GGTCAACAAATCATAAAGATATTGG-3′ and C02198: 5′-TAAACTTCAGGGTGACCAAAAAATCA-3′ [52], and were sequenced with Sanger sequencing method by Tsingke Biotech (Tsingke Biotechnology Co., Ltd., Chengdu, China). We acquired the best-fit and targeted mitochondrial scaffolds by BLAST searches above at least 98%.

### 2.2. Mitochondrial Genome Sequencing, Assembly, Annotation

The mitogenome sequences were obtained on the Illumina Hiseq 2500 platform with 150 bp paired-end reads at Novogene company, Chengdu, China. All the libraries were prepared with an average insert size of 350 bp. Each sample was generated about 10 Gb of raw data which was performed read quality control with FastQC [53] and filtered out low quality reads, adapter contamination and ambiguous bases with Trimmomatic [54]. The obtained clean data was used to assemble and annotate the mitochondrial genome through MitoZ [55]. Gene boundaries were further confirmed and aligned against the published mitogenome sequences of moths using MEGA X [56].

### 2.3. Comparative Analysis of Mitogenome

The overlapping regions and intergenic spacers between genes were counted manually. The base composition and the relative synonymous codon usage were obtained using PhyloSuite [57]. The nucleotide compositional differences between genes were calculated using the formula: AT-skew = (A − T)/(A + T) and GC-skew = (G − C)/(G + C) [58].

### 2.4. Phylogenetic Analysis

Mitochondrial genomes of 66 moths representing six families of Lepidoptera were selected for phylogeny reconstruction, including the nineteen newly sequenced mitogenomes (Table S1). *Xanthochlorus tibetensis* and *Drosophila melanogaster* of Diptera order were selected as outgroups. Thirteen protein-coding genes (PCGs) of these mitogenomes were used as the dataset to construct BI and ML phylogenetic trees by a set of softwares integrated in the PhyloSuite program [57]. Each gene was extracted in batches and then aligned individually by codon-based multiple alignments using the MAFFT algorithm [59] with the L-INS-i strategy and default setting. The conserved regions were identified and unreliably aligned sequences within the datasets were eliminated using Gblock [60]. Then the resulting alignments were concatenated into a single data matrix by PhyloSuite.

Potential substitution saturation was assessed by Xia’s test and index of substitution saturation (Iss) with a GTR model as implemented in DAMBE [61]. Evolutionary rate of PCGs aligned in advance and the ratio of Ka (nonsynonymous substitution rate) and Ks (synonymous substitution rate) were calculated by DnaSP [62].

Subsequently, PartitionFinder [63] was used to infer the optimal partitions and the Bayesian information criterion (BIC) was employed to select the best models under the ‘greedy’ search with linked branch lengths. Phylogenetic trees inferred were constructed using maximum likelihood (ML) with IQ-TREE [64] and Bayesian inference (BI) with MrBayes [65]. For ML analyses, the ultrafast 1000 replicate bootstrapping was conducted in IQ-TREE, and substitutional models were selected with the “Auto” option. BI analyses were executed with 10 million generations with 4 chains, sampling every 1000 generations with a burn in of 25% of sampled values.

## 3. Results and Discussion

### 3.1. Mitogenome Structure and Organization

**Figure 1:**
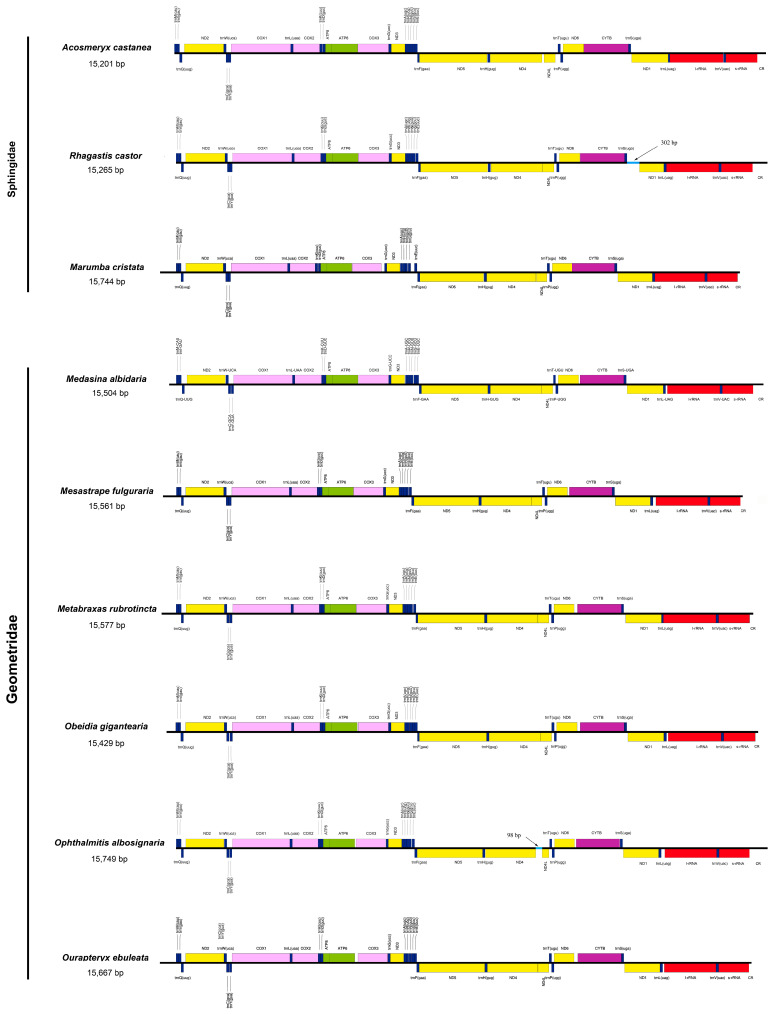

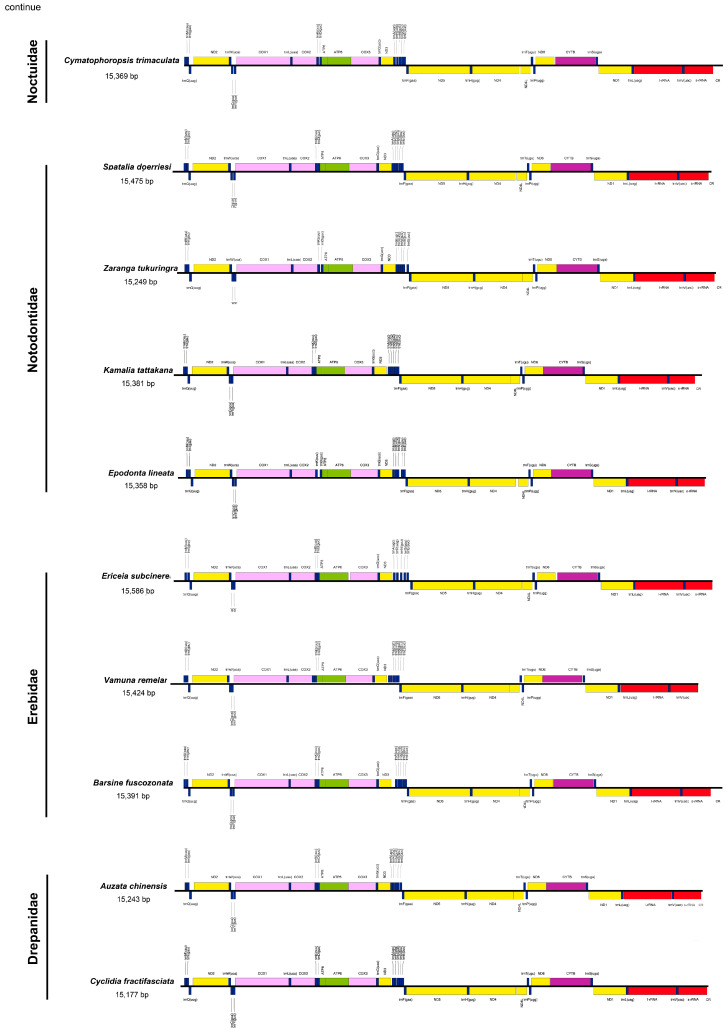
Organizational maps of the nineteen newly sequenced mitogenomes in this study. The genes and intergenic spacers are scaled to their length in the mitogenome. Abbreviations: *COX1*, *COX2*, *COX3*, cytochrome oxidase subunits I, II, III; *CYTB*, cytochrome b apoenzyme; *ND 1–6*, *4L*, NADH dehydrogenase subunits 1–6, 4L; *ATP6*, *ATP8*, ATP synthase subunits 6, 8; l-rRNA, large ribosomal subunit; s-rRNA, small ribosomal subunit; CR, the putative control region; all transfer RNA genes and the corresponding codon are listed.

The newly sequenced moths were involved in six families of Lepidoptera. All of them were assembled as a nearly complete mitogenome. These mitogenome sequences ranged from 15,177 bp (*Cyc. fractifasciata*) to 15,749 bp (*Op. albosignaria*) in length, and each of them included all core 37 mitochondrial genes (13 PCGs, 22 tRNAs genes and two rRNA genes) and the partial control region. Same as most insects, a total of 14 genes including four PCGs, eight tRNAs and two rRNAs were located on the light strand, and the other 23 genes including nine PCGs and 14 tRNAs were located on the height strand (Figure 1) [36,66,67]. The detailed gene information about the nineteen moths was listed in Table S2.

The overall nucleotide composition of all the nineteen months was biased toward A and T, as a common characteristic existing in Lepidoptera insects [48,68]. The total A + T content showed a highest level of 81.9% in *Barsine fuscozonata,* and *Op. albosignaria* while showed a lowest level of 78.1% in *Kamalia tattakana* based on the whole mitogenome sequence (Table 1). However, these values were probably underestimated because of partial AT rich region assembled.

**Figure 2:**
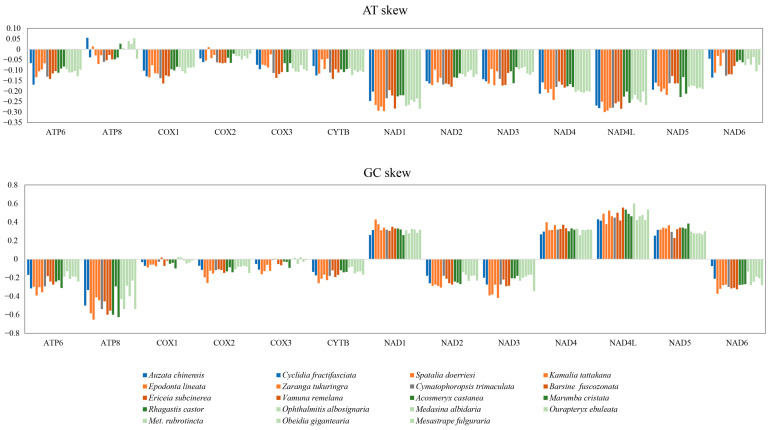
AT and GC skew on individual PCG gene in 19 moth mitogenomes.

Considering the PCGs forming the most mitogenome sequences, we analyzed the strand bias of nucleotide composition of 13 PCGs that are routinely measured by AT skews and GC skews [58]. For all codon sites of the PCGs sequences, it appeared that these moths were almost characterized by significant negative values for AT skews, indicating a strand compositional bias characterized by an excess of A relative to T nucleotides (Table 2). The origin of the strand bias can be related to asymmetric mutational constraints involving deaminations of A and C nucleotides during the replication and/or transcription processes that would result in pairings with C and A, respectively [69]. However, the most taxa exhibited weakly positive GC skew values (<0.1) though the third position of codons showed high negative values. This result was not consistent with the previous view on three Lepidoptera insects [70]. Therefore, we performed a further analysis for each PCG individually to trace the source of an asymmetric base composition. As Figure 2 showed, the positive values of GC skew were mainly from *ND1, ND4, ND4L and ND5*, which was thought to be related to the direction of replication. The variation of GC skew pattern between various taxa still remains unknown. The newly sequenced moth mitogenomes would provide further insight for the evolution of the mitogenome and ecological adaption [71].

### 3.2. Overlapping Sequences and Intergenic Spacers

Gene arrangements in mitogenomes are important evolutionary events and provide valuable phylogenetic signals [72]. As shown in Figure 1, the same gene order of *trnM-trnI-trnQ* was observed among the nineteen mitogenomes of moths, which differed from those of ancestral insects *trnI-trnQ-trnM* [39]. The rearrangement of *trnQ* is considered as a Lepidoptera-specific pattern [70,73,74], which could be resulted from the tandem duplication-random loss (TDRL) [75]. This rearrangement is highly conversed in many taxonomic groups of Lepidoptera such as Erebidae [41,73,76], Notodontidae [74], Limacodidae [36] and so on. While two non-ditrysian species of Hepialoidea in Lepidoptera that had diverged at Early Cretaceous Epoch of the Mesozoic Era displayed an ancestral gene arrangement, evidencing that this gene arrangement likely occurred after that Hepialoidea diverged from other Lepidopteran lineages [77]. As more mitogenomes resources revealed, more gene arrangement-related variations were found in Lepidoptera [78] and other various insect orders [39], which would promote further understandings on the insect evolution, phylogeny and genetics.

The presence of the overlapping region represents a way to economize the design of the mitogenome [79]. For each mitogenome of these 19 moths there were 7 to 13 overlapping regions, and the total length of overlapping sequences ranged from 27 bp (*Ou. ebuleata*) to 72 bp (*Ep. lineata*). The longest overlap (25 bp) was located between *trnL* and *rrnL*, which was consistent with previous studies [80]. The overlaps between *ATP6* and *ATP8,* and between *trnC* and *trnW* were detected in all the 19 moths, and they were reported conserved in Lepidoptera [36,41,67,73,76].

Various intergenic spacers occurred in moths such as regions between *trnQ* and *NAD2*, between *CYTB* and *NAD6*, and between *trnS2* and *NAD1*, [36,41,67,73,76]. Minimizing intergenic spacers is another way to shorten the mitogenome [79]. The large intergenic spacer is little known in moths. To date, the longest intergenic regions (222 bp between *trnE* and *trnF*) have been found in the mitogenome of *Adoxophyes honmai* of Tortricidae [81]. Here, we found a region of 100 bp intergenic spacer between *trnS* and *trnE* in *M. cristata.*

### 3.3. Codon Usage and Contrasting Rates of Evolution

Apart from NAD1 and *COX1*, almost all genes of 19 mitogenomes were initiated with typical ATN codons. In three of six Geometridae species *NAD1* started with TTG, which was reported to be conserved in beetles [82]. TTG was proposed as start codon for economic evolution by minimizing the intergenic space and avoiding overlap with the abutting tRNA [82]. *COX1* possessed diverse starting codons including additional CGA, AAG and AAA and these codons scattered among families, which indicated no relation with lineages. For the stop codons, six PCGs (*ATP6*, *ATP8*, *COX3*, *NAD2*, *NAD6* and *CYTB*) terminated with complete codon of TAA in all 19 mitogenomes, and three PCGs (*NAD1*, *NAD3, NAD4L*) also terminated with “TAG” in several species. Additionally, truncated termination codon “T” or “TA” is common in insects, which could be recognized by endonucleases during polycistronic pre-mRNA transcription [41].

**Figure 3:**
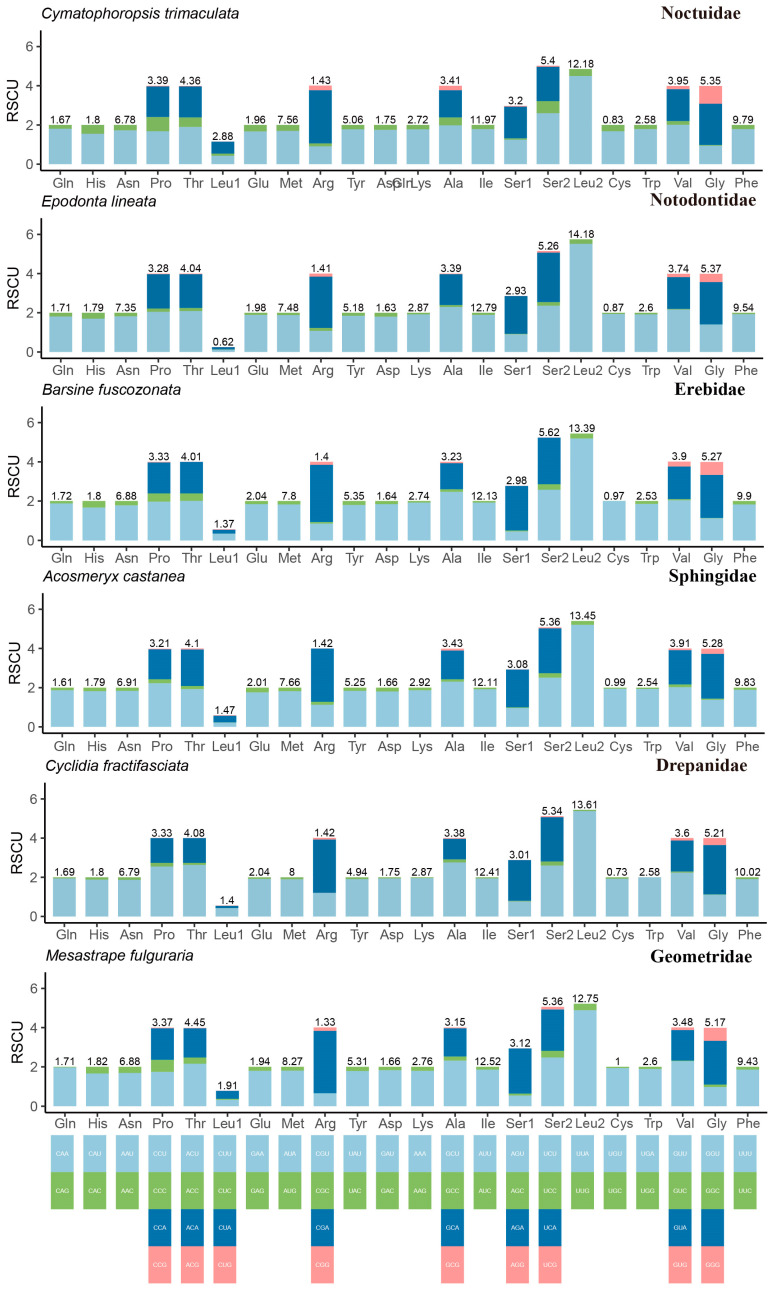
Relative synonymous codon usage (RSCU) in protein-coding genes (PCGs) in the mitogenomes of the nineteen species. Codon families are indicated in boxes below the x-axis; the colors correspond to the stacked columns, and values on the top of the bars denote amino acid usage.

The relative synonymous codon usage (RSCU) of 13 PCGs in the nineteen mitogenomes was calculated (Figure S1). The RSCU values of six species representing six families individually were presented in Figure 3. In general, RSCU showed a similar distribution among families/species. Most amino acids showed a bias on the usage of synonymous codons with a higher frequency of AT than GC, a conserved feature in an insect. The UUA presented significantly high RSCU values (about five) than other codon families (approximately two-fold higher than the second), which was consistent in the six lineages. Whereas, the Notodontidae species showed slightly lower RSCU values. All the 62 codons found were only present in *K. tattakana*. The number of lacked codons ranged from 1 to 6, and the codon AGG, CUG and ACG were absent in multiple species. Five codon families with most usage frequencies (>200) were observed in all nineteen mitogenomes including Leu 2 (UUA), Ile (AUU), Phe (UUU), Met (AUA), Asn (AAU). The distribution pattern of codon families was mostly in keep with previous reports of moths [36,73].

**Figure 4:**
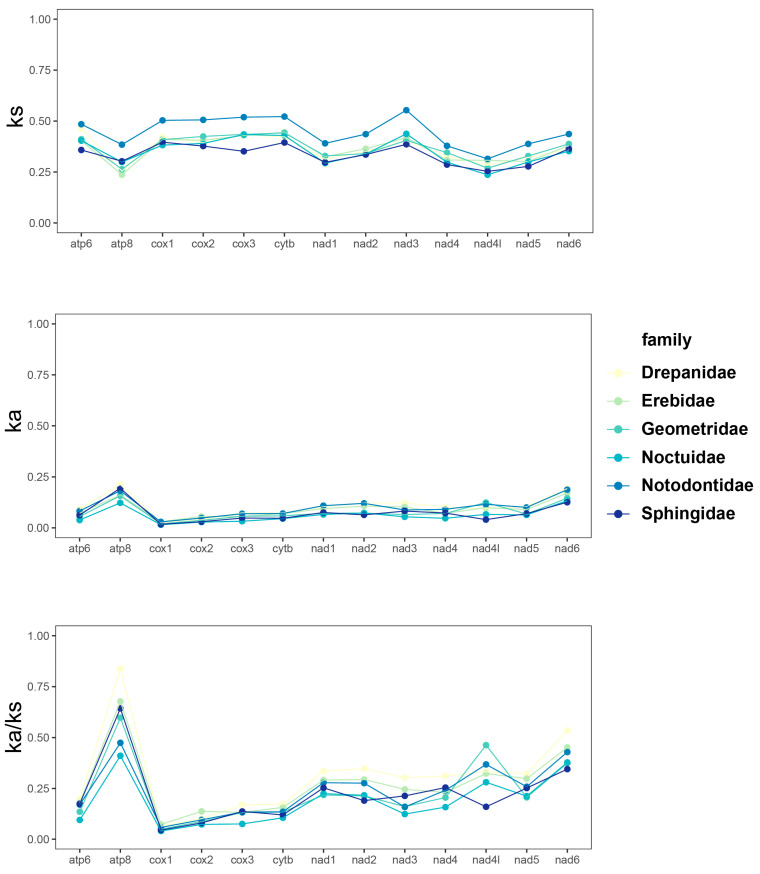
Synonymous and non-synonymous substitutional rates and the ratios of Ka/Ks of protein coding genes. Ka, non-synonymous substitutional rate; Ks, synonymous substitutional rate.

Amino acid sequences are less effected by random similarity and alignment ambiguity compared with nucleotide data [47]. An evolutionary pattern was analyzed among the aligned amino acids sequence of 13 PCGs in six families respectively (including extensively published 57 species) (Figure 4). Ks showed diversity among genes but exhibited similarity among families, with an exception of the Notodontidae family that showed higher Ks than other taxa across all 13 PCGs. Synonymous substitutions are often assumed to be free of selection at the protein level. Notodontidae exhibited relatively higher values than others, indicating an approximation of the neutral mutation rate. Meanwhile, the ratio of Ka/Ks, a typical indicator of evolutionary rate [83,84], did not show similarly elevated values as Ks between Notodontidae and other families. Notodontidae and other moths displayed low evolutionary rates (Ka/Ks < 1) in the 13 PCGs, suggesting that these genes experienced purifying selection. Among them, *ATP8* showed the highest ratio of Ka/Ks, suggesting its least selection pressure and fastest evolution, and *COX1* showed the lowest, suggesting the opposite pattern [66,83,84]. Drepanidae showed higher Ka/Ks values between *ATP8* and *NAD6* than other families, possibly indicating their important role in the evolution of mitogenome.

**Figure 5:**
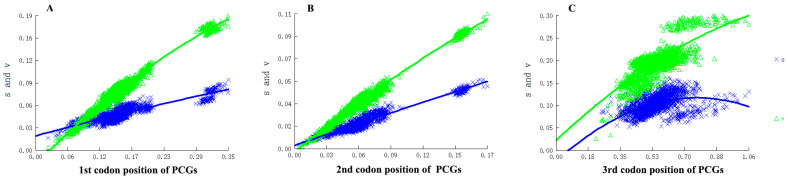
Nucleotide substitution saturation plots of all 13 mitochondrial protein-coding genes. (**A**) The 1st codon positions; (**B**) The 2nd codon positions; (**C**) The 3rd codon positions. Plots in blue and green indicate transition and transversion, respectively.

The saturation plots showed that there was no significant saturation in all codon positions including third codon positions with relatively ‘freely’ evolution, implying that the nucleotides for the phylogenetic reconstruction were qualified (Figure 5).

### 3.4. Phylogenetic Analysis

**Figure 6:**
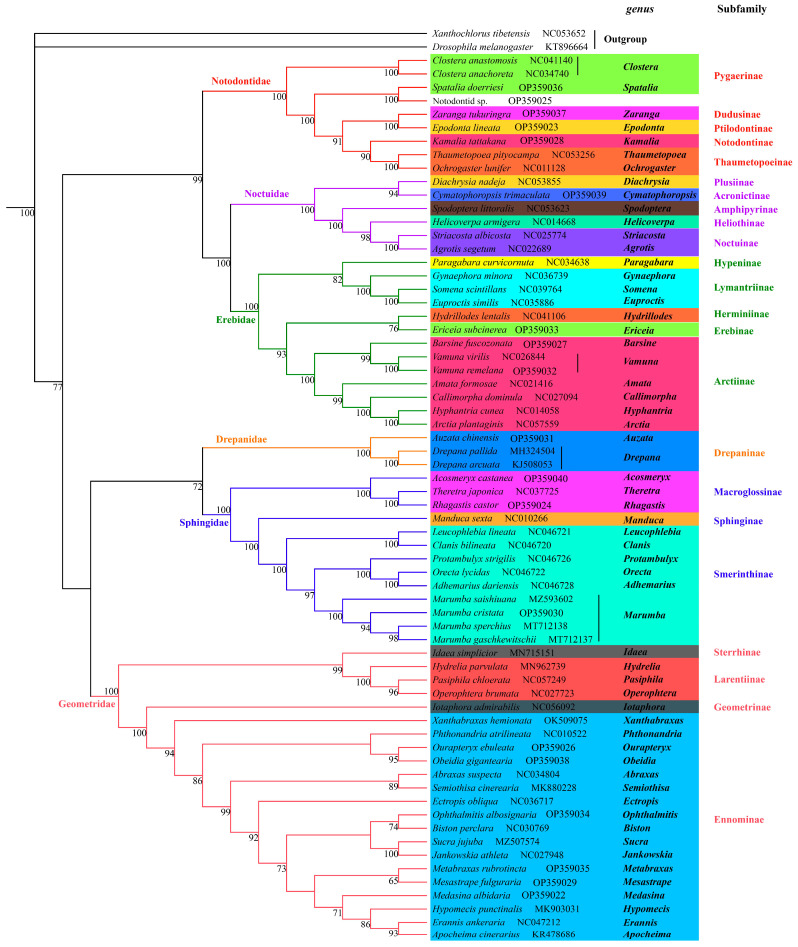
Phylogenetic tree inferred from nucleotide sequences of 13 PCGs using the ML analysis. Numbers on the branches are ML bootstrap support.

**Figure 7:**
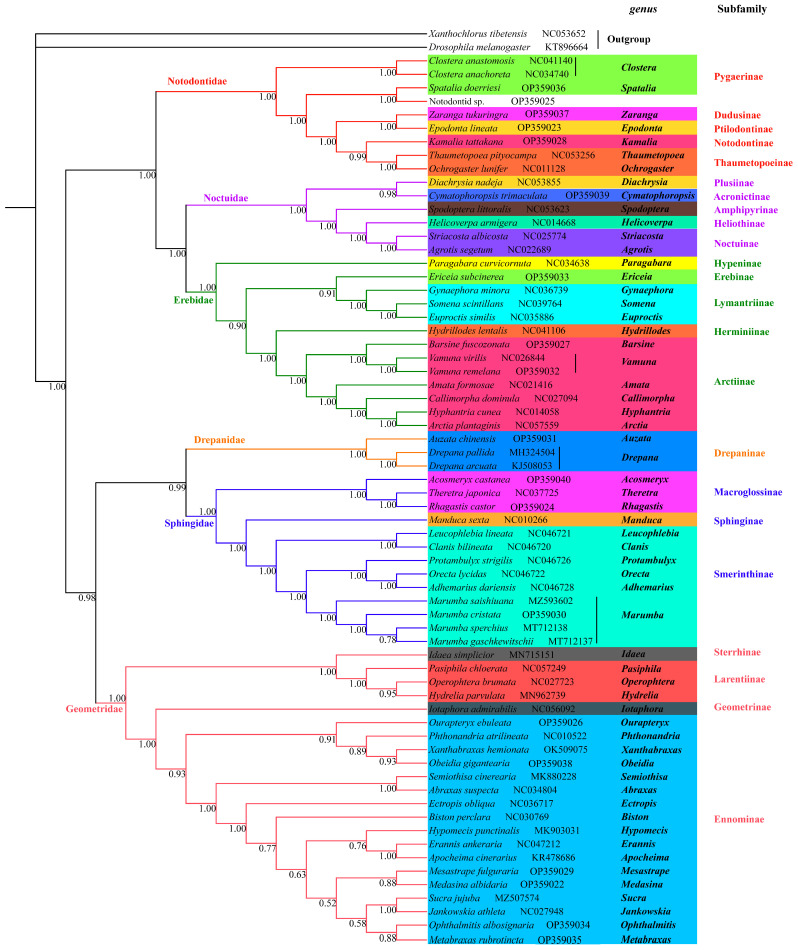
Phylogenetic tree inferred from nucleotide sequences of 13 PCGs using the BI analysis. Numbers on the branches are BI posterior probability.

The phylogenetic analyses were performed based on concatenated 13 PCGs from 66 complete or near complete mitogenomes, representing six families of moths including Drepanidae, Erebidae, Geometridae, Noctuidae, Notodontidae and Sphingidae. Two mitogenomes from Dolichopodidae and Drosophilidae of Diptera, respectively, were used as outgroups. The phylogenetic results generated from Bayesian and ML inferences had mostly identical topologies in the subfamily level, with exceptions of unstable Erebinae and Lymantriinae (Figure 6 and Figure 7).

All species were divided into two major groups.

The first group was composed of noctuids, and the relationship of Notodontidae + (Erebidae + Noctuidae) was well defined with strong supports (PP:1.00; BS:99). They are members of well-accepted six families of Noctuoidea (a superfamily of noctuids) that includes the other three families (Oenosandridae, Euteliidae, Nolidae) unlisted in this study [29]. Noctuoidea shows highly apomorphic morphology and rapid radiation, resulting in a difficulty in addressing the phylogenetic relationship [10,16,29,85]. The phylogenetic results can be impacted bymolecular datasets, taxa samplings and analytical strategies. Zahiri et al. conducted a series of phylogenetic analyses within Noctuoidea using seven nuclear genes and one mitochondrial gene from different sampling sets, and observed different patterns among families [10,16,29,85]. Regier et al. [86] reconstructed a phylogenetic tree with 5–19 genes (6.7–18.6 kb) in 74 noctuoids, and proposed a novel relationship pattern: (Notodontidae + (Erebidae + (Noctuidae + (Euteliidae + Nolidae))). Yang et al. and Zhu et al. reached an agreement based on mitogenome datasets: Notodontidae + (Erebidae + Nolidae + (Euteliidae + Noctuidae)) [74,87]. Despite various patterns within Noctuoidea, our results had shared characteristics with those of studies that Notodontidae originated earlier than Noctuidae and Erebidae; Noctuidae remained diverged with Erebidae.

Within Notodontidae, the Notodontinae that was represented by the newly sequenced genus *Kamalia* clustered with Thaumetopoeinae. This clustering was similar to that of Regier et al., where the representatives of Notodontinae were *Furcula* and *Cerura* [86]. In our study, the sister Ptilodontinae and Dudusiane with representatives of *Epodonta* and *Zaranga,* respectively, were not recovered compared with that of Regier et al. [86], which possibly resulted from different target species. The two genera *Clostera* and *Spatalia* of Pygaerinae formed the basal group while they were separated by three nodes with high bootstrap supports. This finding indicated polyphyletic evolution of Pygaerinae in accordance with that of Schintlmeister [88] and of Regier et al. [86], and we have more reasonable grounds to suspect that *Spatalia* belongs to Pygaerinae. A polygenetic relationship increases the difficulty to understand actual phylogeny among these groups. To resolve this problem requires more sampling involved and wider coverage of taxa. Species of *Cyclidia* were firstly sequenced with mitogenome here. *Cyclidia* belonging to Cyclidiinae of Drepanidae was supported by morphological characters and several gene markers [34,89,90], which was unexpectedly placed in Notodontidae with a strong nodal support based on mitogenome data in our study. We treated it as Notodontid sp. herein, while more mitogenome information of relevant groups was required to resolve the uncertainty.

Within Noctuidae, the relationship of (Amphipyrinae + (Heliothinae + Noctuinae)) + (Plusiinae + Acronictinae) was strongly supported in our study. Previously, Plusiinae as one of the early diverging groups, was separated with these four subfamilies belonging to “higher noctuids”. Heliothinae, Noctuinae and related smaller subfamilies/tribes/unassociated genera formed the “pest clade” [13,14,15,16,27,86]. However, Acronictinae were grouped with Amphipyrinae, rather than Plusiinae in this study. The representative of Amphipyrinae here, *Spodoptera* (Pogue 2002), is unstable in the taxonomic positionthat is initially included in Acronictinae or sometimes in Noctuinae [1,13,91]. *Spodoptera* and allies diverged earlier than Heliothinae, a result identical with that of Regier et al. [86]. However, recent studies suggested the opposite conclusion using more related species within these two subfamilies based on mitogenome datasets [11,73,76,83]. *Cymatophoropsis* (Hampson, 1894), was considered to be one member of Acronictinae in Noctuidae [14] while is was assigned to Erebidae at some taxonomic websites such as NCBI and BOLD, and our result also strongly supportedthe former.

The Erebidae clade consisted of Arctiinae, Lymantriinae and partial concepts of quadrifine noctuoids (those with a strong vein MA2 in the hindwing) that included Herminiinae, Hypeninae and Erebinae [29]. The phylogenetic relationships of Erebidae and its concepts have undergone significant changes, such as these subfamilies in our study formerly as separated families of Noctuidea or as subfamilies of Noctuidae [13,30]. Here, the monophyly of Erebidae was recovered with strong support (PP:100; BS:1.00). The relationships of Hypeninae + ((Erebinae + Lymantriinae) + (Herminiinae + Arctiinae)) were supported by BI tree, by contrast, (Lymantriinae + Hypeninae) + (Arctiinae + (Erebinae + Herminiinae)) was supported by ML tree. Both the molecular and morphological evidences confirmed that Hypeninae was close to the basal lineage to branch off first compared with the other four subfamilies [29,30,41,86]. Previous studies [10,41,86] and our BI tree both supported (PP:1.00) the grouping of Herminiinae and Arctiinae. In trees of Regier et al. [86] and Zahiri et al. [10], these two subfamilies plus Aganainae and Pangraptina were termed as “Arctiine lineage”. The “Arctiine lineage” remained divergent with “Erebine lineage” including Erebinae and additional families such as Scolecocampinae, Boletobinae and Hypenodinae. Lymantriinae, for its unstable position, was included in neither “Arctiine lineage” or “Erebine lineage”. Thus, the topology based on the Bayesian analysis might be closer to the real phylogeny of the Erebinae family. Despite the differences shown in the two trees, the relationships within Arctiinae subfamily appeared to get resolved well. The divergence between *Vamuna* and the other clade (consisting of *Amata*, *Callimorpha*, *Hydrillodes* and *Arctia*) was stable, which was confirmed by Galarza et al. based on eleven mitochondrial genes (excluding *ATP8* and *ND6*) [9].

The second group of Geometridae + (Sphingidae + Drepanidae) was recovered, which suggested that Geometroidea was close to the sister group of Bombycoidea and Drepanoidea, with Noctuoidea as the basal branch. Although increasing studies aimed to resolve the phylogeny of Lepidoptera among superfamilies, accumulated contradictories were observed. For example, a phylogenetic pattern of Lepidoptera was supported by Regier et al. (2013) [27] using 483 taxon for 19 protein-coding nuclear genes: ((Noctuoidea + Bombycoidea) + Geometroidea) + Drepanoidea; a different pattern was observed according to the partial results of Kawahara et al.’s study [1] using 186 species for 2098 orthologous protein-coding genes: ((Geometroidea + Bombycoidea) + Noctuoidea) + Drepanoidea; Heikkilä et al. [92] proposed another pattern combining morphological and molecular data covering 473 taxa and 6702 characters (530 morphological characters; 6172 bp): ((Noctuoidea + Bombycoidea) + Drepanoidea) + Geometroidea. However, our study showed a distinctive pattern with all mentioned above.

The clade of *Auzata* and *Drepana* as representatives of Drepanidae was sister to Sphingidae. Within Sphingidae, there were three subfamilies: Sphinginae, Smerinthinae and Macroglossinae. Each of these was recovered as monophyletic with very high support values (PP = 1.00, BS = 100), while monophyletic Sphinginae required further testing as it comprised only a single genus and species herein. As our topology showed, Sphinginae was sister to Smerinthinae and Macroglossinae was firstly branched off as the basal group, which accorded with the results of Kawahara et al. by five nuclear genes [1] and of Wang et al. by two datasets of PCG123R (including all codon positions) and PCG12R (removing the third codon positions) [93]. The intergroups of Smerinthinae were also well-defined with strong support, and three major groups were recovered herein corresponding to the tribe Leucophlebiini (*Leucophlebia*, *Clanis*), tribe Ambulycini (*Orecta*, *Protambulyx*, *Adhemarius*) and tribe Sichiini (*Marumba*), a result identical with that of Wang et al. [93].

Within Geometridae, two major clades were recovered by BI and ML trees (PP:1.00; BS:100). The first one included Sterrhinae and Larentiinae; the second one included Ennominae and Geometrinae. The sister relationship of Sterrhinae and Larentiinae has been confirmed by morphological evidences [94] and molecular analyses [95,96], but there existed some exceptions that some Larentiinae species were included in [97] or just next to Sterrhinae instead as sister group to Sterrhinae [17]. Ennominae is the largest subfamily of the Geometridae, highly diverse in the morphology [17]. Within Ennominae, many tribes and genera on phylogenetic status have been in dispute or have not been assigned. Ennominae formed the monophyletic group with strong support (PP:0.93; BS:94). To date, almost all molecular studies have reached agreement on the monophyly of Ennominae [17,96,98,99] except Young et al.’s study [100]. Despite weak support at some internal nodes, there were clearly observed two major subclades within Ennominae, corresponding to the two typical divisions of “ennomine” and “boarmiine” moths based on the structure of the cremaster in the pupal stage [101,102]. Two unassigned genera, *Obeidia* and *Xanthabraxas,* showed a close relationship with members of “ennomine” *Phthonandria* (tribe gnophini) and *Ourapteryx* (tribe ourapterygini). In the “boarmiine” subcalde, the sister *Semiothisa* and *Abraxas*, as representatives of tribe macariini and abraxini respectively, together were sisters to the rest of the taxa with strong supports. The rest of the taxa mostly belonged to Boarmiini tribe, with an exception of *Metabraxas* belonging to cystidiini tribe, which was not coincident with monophyletic Boarmiini [17,18]. Cystidiini is a Palaearctic tribe, first introduced by Stekolnikov and Kuznetzov [103] but not included in the Forum Herbulot list of the Geometridae tribes (2014) being little known [104] either morphologically or molecularly. It is the first time that species of cystidiini have been involvedin a molecular analysis based on a whole mitogenome sequence, although *Metabraxas coryneta* was previously reported for species identification with a *COI* gene sequence [52]. *Metabraxas* as the sister group of *Ophthalmitis* was supported by the BI tree (PP:0.88) in contrast with being the sister group of *Mesastrape* which was supported by ML tree (BS:65). Poorly nodal supports in resolving the relationships within Boarmiini may get improved when given good taxon sampling.

## 4. Conclusions

Our study determined nineteen nearly complete mitochondrial genomes across six families of Lepidoptera by next generation sequencing technologies including Drepanidae, Erebidae, Geometridae, Noctuidae, Notodontidae and Sphingidae. The comparative mitogenome analyses suggested conserved characteristics among these species such as being highly A + T-biased, having a rapidly evolved *ATP8* gene, being a typical rearrangement of *trnM-trnI-trnQ* and having a weak strand bias based on GC skew of PCGs. Phylogenetic results supported the relationships at the superfamily level as follows: (Noctuoidea + (Geometroidea + (Bombycoidea + Drepanoidea))); a different pattern from previous reports. The relationships of most families among subordinated taxa were well defined with strong supports such as Drepanidae and Sphingidae, but partial clades within species-rich groups such as Ennominae were inadequate in support. Therefore, robust phylogeny needs further investigation with increased taxa sampling. With expanded comparative analyses on moths, our studies will throw greater light on the evolution and phylogeny of moths and the biological control of pests.

## Figures and Tables

**Table 1:** Nucleotide composition of the 19 mitochondrial genomes of moths.

Family	Species	Mitogenome Length (bp)	A%	T (U)%	C%	G%	AT (%)	GC (%)
Drepanidae	*Auzata chinensis*	15,243	41.1	39.2	11.60	8.20	80.30	19.80
	*Cyc. fractifasciata*	15,177	39.8	40.8	11.90	7.50	80.60	19.40
Erebidae	*B. fuscozonata*	15,391	40.2	41.7	10.70	7.40	81.90	18.10
	*Ericeia subcinerea*	15,586	39.9	40.3	12.40	7.50	80.20	19.90
	*Vamuna remelana*	15,424	40.2	40.2	12.10	7.50	80.40	19.60
Geometridae	*Medasina albidaria*	15,504	41.4	39.8	10.90	7.90	81.20	18.80
	*Mesastrape fulguraria*	15,561	41.7	39.4	11.40	7.40	81.10	18.80
	*Metabraxas rubrotincta*	15,577	41.9	39.5	11.20	7.50	81.40	18.70
	*Obeidia gigantearia*	15,429	41.2	39.9	11.20	7.70	81.10	18.90
	*Op. albosignaria*	15,749	42.1	39.8	10.70	7.40	81.90	18.10
	*Ourapteryx ebuleata*	15,667	41	39.5	11.70	7.80	80.50	19.50
Noctuidae	*Cymatophoropsis trimaculata*	15,369	40.1	40.3	11.80	7.70	80.40	19.50
Notodontidae	*Epodonta lineata*	15,358	40.7	39.3	12.20	7.70	80.00	19.90
	*K. tattakana*	15,381	40.7	37.4	13.80	8.10	78.10	21.90
	*Spatalia doerriesi*	15,475	39.2	39.7	13.50	7.70	78.90	21.20
	*Zaranga tukuringra*	15,249	41.1	37.5	13.60	7.90	78.60	21.50
Sphingidae	*Acosmeryx castanea*	15,201	41	39.6	11.80	7.60	80.60	19.40
	*Marumba cristata*	15,744	40.5	41.1	11.10	7.30	81.60	18.40
	*Rhagastis castor*	15,265	41.1	39.2	12.30	7.50	80.30	19.80

**Table 2:** AT and GC skew at different position of codon for 13 PCG genes in 19 moth mitogenomes.

Species	AT Skew				GC Skew			
	1st	2nd	3rd	123	1st	2nd	3rd	123
*Au. chinensis*	−0.0052	−0.3718	−0.0616	−0.1373	0.2430	−0.0965	−0.1821	0.0303
*Cyc. fractifasciata*	0.0023	−0.3761	−0.0902	−0.1461	0.1934	−0.0988	−0.2555	0.0007
*B. fuscozonata*	−0.0046	−0.3766	−0.0856	−0.1466	0.2484	−0.1102	−0.2340	0.0376
*Er. subcinerea*	−0.0054	−0.3707	−0.0822	−0.1448	0.2408	−0.1057	−0.2493	0.0165
*V. remelana*	−0.0095	−0.3698	−0.0949	−0.1496	0.2451	−0.1096	−0.2853	0.0205
*Med. albidaria*	−0.0126	−0.3665	−0.0660	−0.1388	0.2639	−0.0947	−0.1465	0.0533
*Met. rubrotincta*	0.0002	−0.3622	−0.0647	−0.1331	0.2907	−0.1043	−0.2281	0.0447
*Ma. cristata*	−0.0065	−0.3652	−0.0423	−0.1268	0.2560	−0.0925	−0.2909	0.0351
*Op. albosignaria*	−0.0053	−0.3598	−0.0636	−0.1334	0.2933	−0.0963	−0.1317	0.0634
*Ob. gigantearia*	−0.0224	−0.3607	−0.0606	−0.1371	0.2772	−0.0984	−0.2333	0.0369
*Ou. ebuleata*	−0.0078	−0.3642	−0.0642	−0.1366	0.2732	−0.0921	−0.2059	0.0359
*Cym. trimaculata*	−0.0155	−0.3735	−0.0901	−0.1512	0.2457	−0.0980	−0.2776	0.0203
*Ep. lineata*	−0.0125	−0.3743	−0.0825	−0.1482	0.2350	−0.1044	−0.2757	0.0117
*K. tattakana*	0.0122	−0.3690	−0.0577	−0.1305	0.1908	−0.1142	−0.2283	−0.0228
*S. doerriesi*	−0.0014	−0.3740	−0.0994	−0.1522	0.1919	−0.0971	−0.2088	−0.0049
*Mes. fulguraria*	−0.0078	−0.3613	−0.0792	−0.1400	0.2594	−0.1028	−0.2616	0.0204
*Z. tukuringra*	0.0062	−0.3739	−0.0776	−0.1410	0.1906	−0.1063	−0.2044	−0.0056
*Ac. castanea*	−0.0215	−0.3668	−0.0582	−0.1381	0.2508	−0.1036	−0.1589	0.0383
*Ma. cristata*	−0.0065	−0.3652	−0.0423	−0.1268	0.2560	−0.0925	−0.2909	0.0351
*R. castor*	0.0174	−0.3668	−0.0550	−0.1255	0.2039	−0.1048	−0.2125	0.0037

## Data Availability

The data supporting the findings of this study are openly available from the NCBI (https://www.ncbi.nlm.nih.gov/). Accession numbers are listed in Table S1.

## Supplementary Materials

The following supporting information can be downloaded at: https://www.mdpi.com/article/10.3390/genes13091634/s1, Figure S1: The relative synonymous codon usage (RSCU) of 13 PCGs in the nineteen mitogenomes. Table S1: Species information of mitochondrion genomes used in the study. Table S2: Details on the gene organization of the nineteen moth mitogenomes.
