# Supplementary material for: Enhanced Resolution of Evolution and Phylogeny of the Moths Inferred from Nineteen Mitochondrial Genomes
Source: Genes (Basel). 2022 Sep 12;13(9):1634. doi: 10.3390/genes13091634 (PMC9498458; doi:10.3390/genes13091634)
Supplement: Supplementary file 1 [file genes-13-01634-s001.zip › Figure S1.pdf]

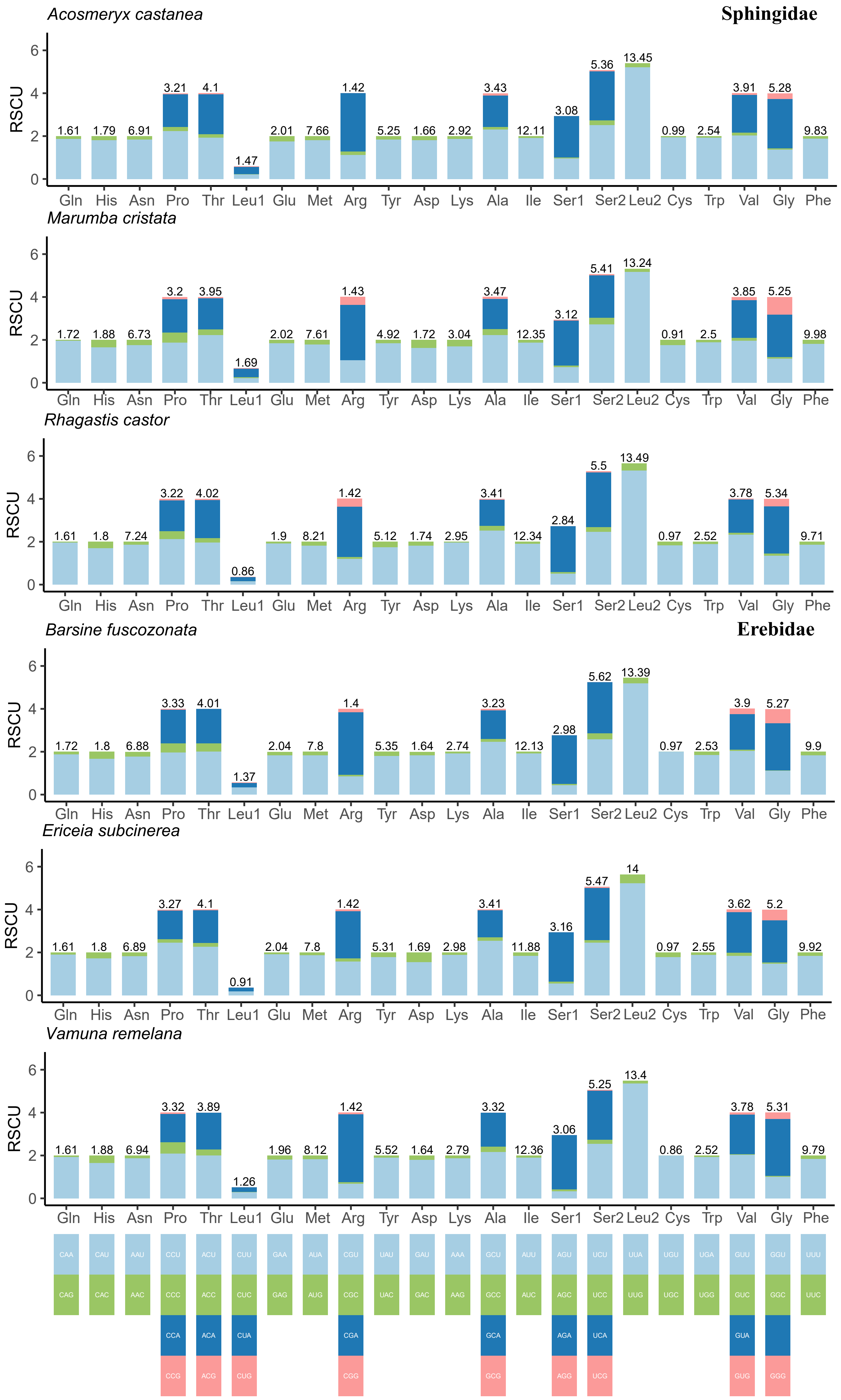
Acosmeryx castanea
Sphingidae
RSCU
1.61 1.79 6.91 3.21 4.1 1.47 2.01 7.66 1.42 5.25 1.66 2.92 3.43 12.11 3.08 5.36 13.45 0.99 2.54 3.91 5.28 9.83
Gln His Asn Pro Thr Leu1 Glu Met Arg Tyr Asp Lys Ala Ile Ser1 Ser2 Leu2 Cys Trp Val Gly Phe
Marumba cristata
RSCU
1.72 1.88 6.73 3.2 3.95 1.69 2.02 7.61 1.43 4.92 1.72 3.04 3.47 12.35 3.12 5.41 13.24 0.91 2.5 3.85 5.25 9.98
Gln His Asn Pro Thr Leu1 Glu Met Arg Tyr Asp Lys Ala Ile Ser1 Ser2 Leu2 Cys Trp Val Gly Phe
Rhagastis castor
RSCU
1.61 1.8 7.24 3.22 4.02 0.86 1.9 8.21 1.42 5.12 1.74 2.95 3.41 12.34 2.84 5.5 13.49 0.97 2.52 3.78 5.34 9.71
Gln His Asn Pro Thr Leu1 Glu Met Arg Tyr Asp Lys Ala Ile Ser1 Ser2 Leu2 Cys Trp Val Gly Phe
Barsine fuscozonata
Erebidae
RSCU
1.72 1.8 6.88 3.33 4.01 1.37 2.04 7.8 1.4 5.35 1.64 2.74 3.23 12.13 2.98 5.62 13.39 0.97 2.53 3.9 5.27 9.9
Gln His Asn Pro Thr Leu1 Glu Met Arg Tyr Asp Lys Ala Ile Ser1 Ser2 Leu2 Cys Trp Val Gly Phe
Ericeia subcinerea
RSCU
1.61 1.8 6.89 3.27 4.1 0.91 2.04 7.8 1.42 5.31 1.69 2.98 3.41 11.88 3.16 5.47 14 0.97 2.55 3.62 5.2 9.92
Gln His Asn Pro Thr Leu1 Glu Met Arg Tyr Asp Lys Ala Ile Ser1 Ser2 Leu2 Cys Trp Val Gly Phe
Vamuna remelana
RSCU
1.61 1.88 6.94 3.32 3.89 1.26 1.96 8.12 1.42 5.52 1.64 2.79 3.32 12.36 3.06 5.25 13.4 0.86 2.52 3.78 5.31 9.79
Gln His Asn Pro Thr Leu1 Glu Met Arg Tyr Asp Lys Ala Ile Ser1 Ser2 Leu2 Cys Trp Val Gly Phe
CAA CAU AAU CCU ACU CUU GAA AUA CGU UAU GAU AAA GCU AUU AGU UCU UUA UGU UGA GUU GGU UUU
CAG CAC AAC CCC ACC CUC GAG AUG CGC UAC GAC AAG GCC AUC AGC UCC UUG UGC UGG GUC GGC UUC
CCA ACA CUA CGA GCA AGA UCA GUA
CCG ACG CUG CGG GCG AGG UCG GUG GGG

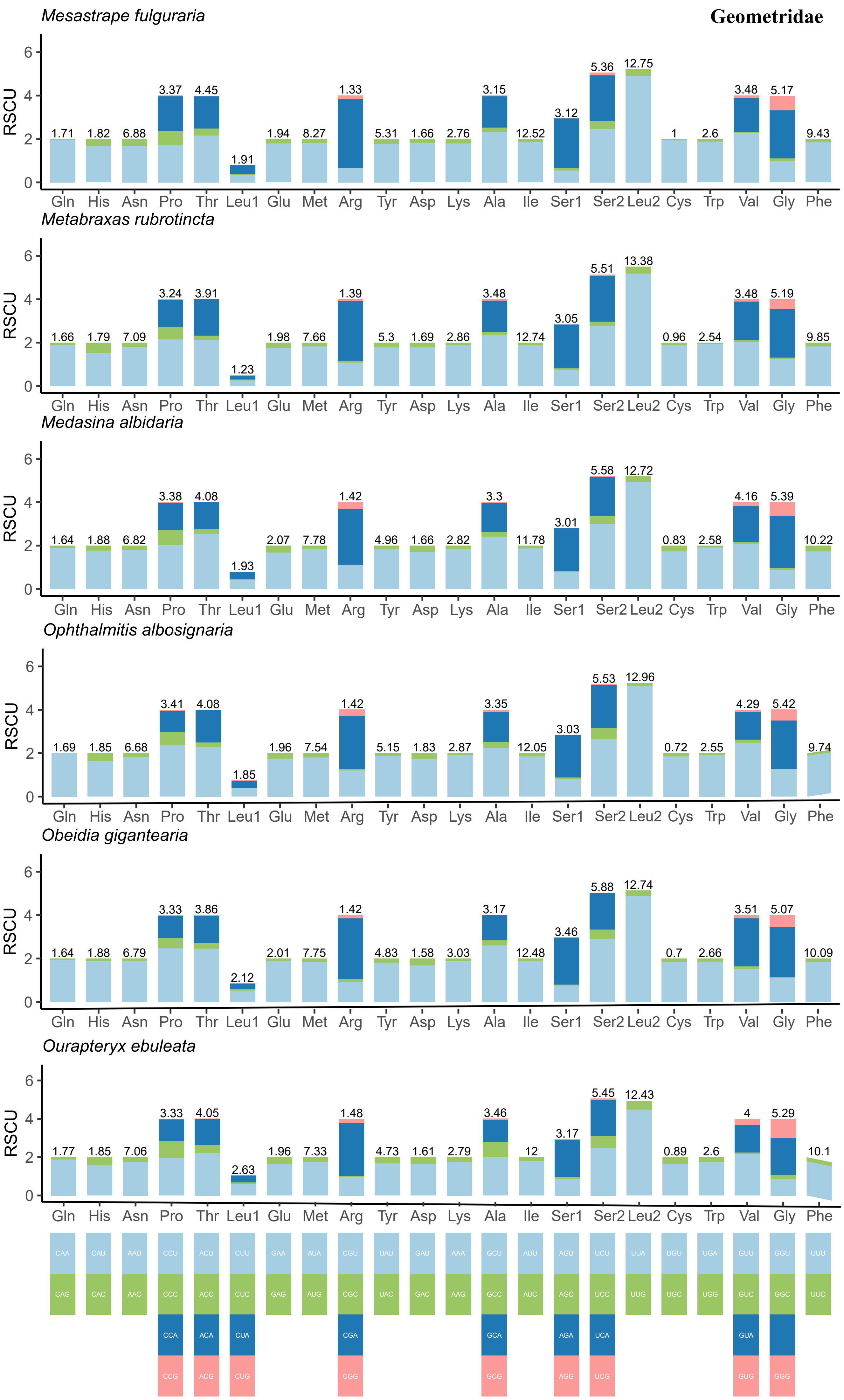
Geometridae
RSCU
Mesastrape fulguraria
1.71 1.82 6.88 3.37 4.45 1.91 1.94 8.27 1.33 5.31 1.66 2.76 3.15 12.52 3.12 5.36 12.75 1 2.6 3.48 5.17 9.43
Metabraxas rubrotincta
1.66 1.79 7.09 3.24 3.91 1.23 1.98 7.66 1.39 5.3 1.69 2.86 3.48 12.74 3.05 5.51 13.38 0.96 2.54 3.48 5.19 9.85
Medasina albidaria
1.64 1.88 6.82 3.38 4.08 1.93 2.07 7.78 1.42 4.96 1.66 2.82 3.3 11.78 3.01 5.58 12.72 0.83 2.58 4.16 5.39 10.22
Ophthalmitis albosignaria
1.69 1.85 6.68 3.41 4.08 1.85 1.96 7.54 1.42 5.15 1.83 2.87 3.35 12.05 3.03 5.53 12.96 0.72 2.55 4.29 5.42 9.74
Obeidia gigantearia
1.64 1.88 6.79 3.33 3.86 2.12 2.01 7.75 1.42 4.83 1.58 3.03 3.17 12.48 3.46 5.88 12.74 0.7 2.66 3.51 5.07 10.09
Ourapteryx ebuleata
1.77 1.85 7.06 3.33 4.05 2.63 1.96 7.33 1.48 4.73 1.61 2.79 3.46 12 3.17 5.45 12.43 0.89 2.6 4 5.29 10.1
0 2 4 6
Gln His Asn Pro Thr Leu1 Glu Met Arg Tyr Asp Lys Ala Ile Ser1 Ser2 Leu2 Cys Trp Val Gly Phe
CAA CAU AAU CCU ACU CUU GAA AUA CGU UAU GAU AAA GCU AUU AGU UCU UUA UGU UGA GUU GGU UUU
CAG CAC AAC CCC ACC CUC GAG AUG CGC UAC GAC AAG GCC AUC AGC UCC UUG UGC UGG GUC GGC UUC
CCA ACA CUA CGA GCA AGA UCA GUA
CCG ACG CUG CGG GCG AGG UCG GUG GGG

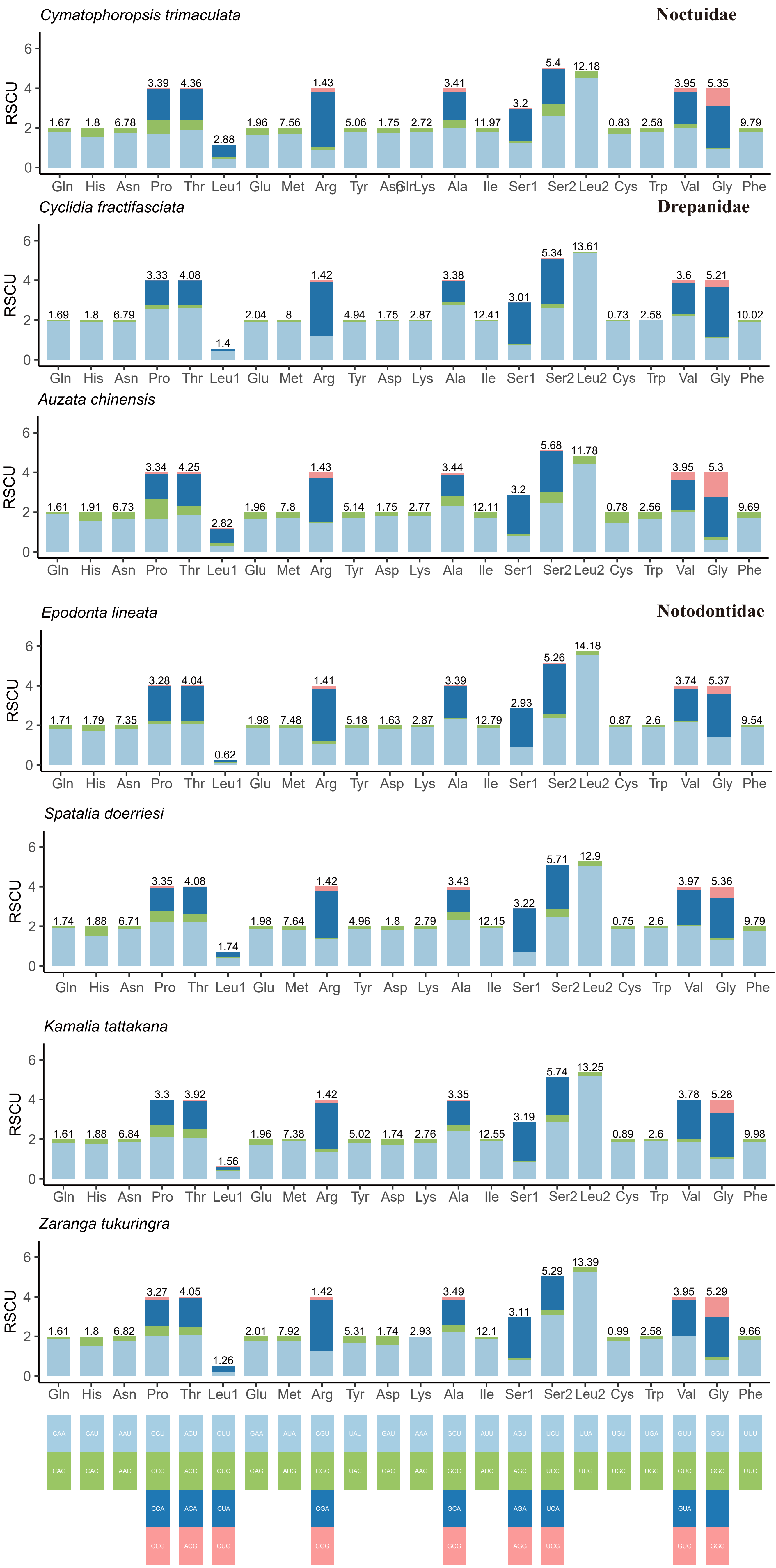
Cymatophoropsis trimaculata
Noctuidae
RSCU
1.67 1.8 6.78 3.39 4.36 2.88 1.96 7.56 1.43 5.06 1.75 2.72 3.41 11.97 3.2 5.4 12.18 0.83 2.58 3.95 5.35 9.79
Gln His Asn Pro Thr Leu1 Glu Met Arg Tyr Asp Gln Lys Ala Ile Ser1 Ser2 Leu2 Cys Trp Val Gly Phe
Cyclidia fractifasciata
Drepanidae
RSCU
1.69 1.8 6.79 3.33 4.08 1.4 2.04 8 1.42 4.94 1.75 2.87 3.38 12.41 3.01 5.34 13.61 0.73 2.58 3.6 5.21 10.02
Gln His Asn Pro Thr Leu1 Glu Met Arg Tyr Asp Lys Ala Ile Ser1 Ser2 Leu2 Cys Trp Val Gly Phe
Auzata chinensis
RSCU
1.61 1.91 6.73 3.34 4.25 2.82 1.96 7.8 1.43 5.14 1.75 2.77 3.44 12.11 3.2 5.68 11.78 0.78 2.56 3.95 5.3 9.69
Gln His Asn Pro Thr Leu1 Glu Met Arg Tyr Asp Lys Ala Ile Ser1 Ser2 Leu2 Cys Trp Val Gly Phe
Epodonta lineata
Notodontidae
RSCU
1.71 1.79 7.35 3.28 4.04 0.62 1.98 7.48 1.41 5.18 1.63 2.87 3.39 12.79 2.93 5.26 14.18 0.87 2.6 3.74 5.37 9.54
Gln His Asn Pro Thr Leu1 Glu Met Arg Tyr Asp Lys Ala Ile Ser1 Ser2 Leu2 Cys Trp Val Gly Phe
Spatalia doerriesi
RSCU
1.74 1.88 6.71 3.35 4.08 1.74 1.98 7.64 1.42 4.96 1.8 2.79 3.43 12.15 3.22 5.71 12.9 0.75 2.6 3.97 5.36 9.79
Gln His Asn Pro Thr Leu1 Glu Met Arg Tyr Asp Lys Ala Ile Ser1 Ser2 Leu2 Cys Trp Val Gly Phe
Kamalia tattakana
RSCU
1.61 1.88 6.84 3.3 3.92 1.56 1.96 7.38 1.42 5.02 1.74 2.76 3.35 12.55 3.19 5.74 13.25 0.89 2.6 3.78 5.28 9.98
Gln His Asn Pro Thr Leu1 Glu Met Arg Tyr Asp Lys Ala Ile Ser1 Ser2 Leu2 Cys Trp Val Gly Phe
Zaranga tukuringra
RSCU
1.61 1.8 6.82 3.27 4.05 1.26 2.01 7.92 1.42 5.31 1.74 2.93 3.49 12.1 3.11 5.29 13.39 0.99 2.58 3.95 5.29 9.66
Gln His Asn Pro Thr Leu1 Glu Met Arg Tyr Asp Lys Ala Ile Ser1 Ser2 Leu2 Cys Trp Val Gly Phe
CAA CAU AAU CCU ACU CUU GAA AUA CGU UAU GAU AAA GCU AUU AGU UCU UUA UGU UGA GUU GGU UUU
CAG CAC AAC CCC ACC CUC GAG AUG CGC UAC GAC AAG GCC AUC AGC UCC UUG UGC UGG GUC GGC UUC
CCA ACA CUA CGA GCA AGA UCA GUA
CCG ACG CUG CGG GCG AGG UCG GUG GGG
